# The late Archaean to early Proterozoic origin and evolution of anaerobic methane‐oxidizing archaea

**DOI:** 10.1002/mlf2.12013

**Published:** 2022-03-30

**Authors:** Yinzhao Wang, Ruize Xie, Jialin Hou, Zhenbo Lv, Liuyang Li, Yaoxun Hu, Hungchia Huang, Fengping Wang

**Affiliations:** ^1^ State Key Laboratory of Microbial Metabolism, School of Life Sciences and Biotechnology Shanghai Jiao Tong University Shanghai China; ^2^ School of Oceanography Shanghai Jiao Tong University Shanghai China; ^3^ Southern Marine Science and Engineering Guangdong Laboratory (Zhuhai) Zhuhai China

## Abstract

Microorganisms, called anaerobic methane‐oxidizing archaea (ANME), can reduce a large amount of greenhouse gas methane and therefore have the potential to cool the Earth. We collected nearly all ANMEs genomes in public databases and performed a comprehensive comparative genomic analysis and molecular dating. Our results show that ANMEs originated in the late Archaean to early Proterozoic eon. During this period of time, our planet Earth was experiencing the Great Oxygenation Event and Huronian Glaciation, a dramatic drop in the Earth's surface temperature. This suggests that the emergence of ANMEs may contribute to the reduction of methane at that time, which is an unappreciated potential cause that led to the Huronian Glaciation.

Methanogenesis under anaerobic conditions is considered one of the most ancient biogeochemical pathways on Earth^1^, ^2^, ^3^, ^4^, and several studies hypothesized that the archaeal ancestor might also conduct this reaction^5^, ^6^, which dated back to the late Hadean or early Archaean eon^7^, ^8^. Reactions within the methanogenesis pathway can also be reversed^9^, which is known as anaerobic oxidation of methane (AOM) and is carried out by anaerobic methane‐oxidizing archaea (ANME). It was estimated that the AOM process is distributed globally and removes over 80% of methane within anaerobic sedimentary environments in the modern ocean^10^. The ANMEs contribute to the control of methane emissions in both marine and terrestrial ecosystems, preventing potential global warming effects^2^, ^10^. Undoubtedly, the historical emergence and thriving of ANME in the Earth ecosystems could have caused a remarkable reduction in methane emissions and a drastic global carbon imbalance^11^. However, although both geological and molecular dating evidences for the origin of methanogen has been found and adequately discussed^7^, ^8^, ^12^, ^13^, few such implications have been provided for ANME.

Currently, all ANMEs are phylogenetically affiliated with the superphylum Euryarchaeota from the domain Archaea^4^. They belong to the order *Ca*. Methanophagales (previously named ANME‐1) and Methanosarcinales (including four major lineages: ANME‐2a/b, ANME‐2c, ANME‐2d, and ANME‐3). ANME‐1 was first discovered in cold seep samples and then was found in a wide range of habitats, including marine and freshwater ecosystems^4^, ^9^, ^14^. The ANME‐2a/b lineages usually dominate most methane seep sediments, and the ANME‐2c clade occupies similar ecological niches^9^, ^15^. The *Ca*. Methanoperedenaceae (previously named ANME‐2d) are also closely related to ANME‐2a/b in the phylogenetic tree, but they are able to live without partner bacteria and are largely derived from freshwater habitats^16^. The ANME‐3 clade is usually dominant in methane‐rich mud volcanoes^17^. In this study, we conducted systematic comparative genomic analyses of all available ANMEs genomes from the National Center of Biotechnology Information (NCBI) prokaryotic genome database, including a potential ANME‐3 genome, and revealed the origin and evolutionary history of ANMEs.

The critical biochemical reaction of methanogenesis and AOM is the reduction of methyl‐coenzyme M (CoM) and activation of methane, respectively, via a conserved enzyme methyl‐CoM reductase (MCR, usually with three subunits McrABG). The gene coding for alpha subunit of MCR (McrA) is considered a phylogenetic marker gene for both methanogens and ANMEs^2^. For phylogenetic analyses, we downloaded all potential ANMEs genomes, as well as the representative euryarchaeal methanogens genomes from the NCBI genome database (File S1 and Tables S1–S3). Phylogenetic trees were constructed based on the protein sequences of concatenated, conserved single‐copy marker genes (genome, Figure 1A) and McrABG‐encoding genes (Figure 1B). In total, there are 21 ANME‐1 genomes (completeness, 70.72%–94.39%; redundancy, 0.93%–6.54%); 38 ANME‐2 genomes (completeness, 71.17%–100%; redundancy, 0%–6.39%); and one potential ANME‐3 genomes (completeness, 61.61%; redundancy, 6.85%). All the ANMEs belong to the Euryarchaeota superphylum and are classified into two orders. ANME‐1 consists of one order‐level monophyletic group (*Ca*. Methanophagales), and together with the other three orders *Ca*. Alkanophagales, *Ca*. Syntrophoarchaeales, and *Ca*. Santabarbaracales, form the class *Ca*. Syntrophoarchaeia^4^, ^8^. Except for ANME‐1, the other three members within this class are considered anaerobic multicarbon alkane oxidizers, while ANME‐1 is regarded as a strict methane oxidizer. The deep‐branching lineages from the class *Ca*. Syntrophoarchaeia are all capable of degrading short‐chain alkanes such as *n*‐butane and propane, and the member from the sister clade of ANME‐1 was also proposed to be an alkane degrader^8^ (Figure 1A). These anaerobic alkane degraders use divergent MCRs to activate alkanes under anaerobic environments using the same mechanism with AOM and are thus called alkyl‐CoM reductase (ACR)^4^. All ACR sequences cluster together on the phylogenetic tree (Figure 1B), whereas MCR sequences from ANME‐1 are grouped together with methanogen *Ca*. Methanofastidiosales or the recently reported *Ca*. Nuwarchaeales^8^, suggesting a horizontal gene transfer (HGT) event. The phylogenomic tree of the ANME‐1 clade can be further divided into the basal lineage B22_G9 and the other ANME‐1 clades, similar to the topology of the McrABG/McrAG phylogenetic trees (Figure S1A–C). This indicates that B22_G9 may represent an early evolved ANME‐1 lineage in this order. Both ANME‐1 B22_G9 and its alkane‐degrading sister lineage *Ca*. Alkanophagales were first discovered in the alkane‐rich hydrothermal Guaymas Basin sediment^18^, suggesting that ANME‐1 had a thermophilic origin history.

**Figure 1:**
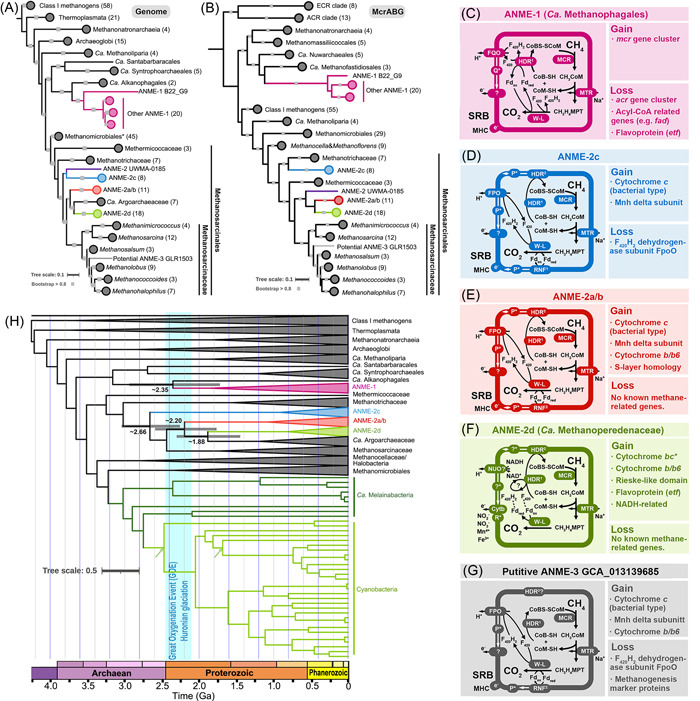
The evolutionary history of different anaerobic methane‐oxidizing archaea (ANME) lineages. (A) Phylogenomic tree of methanogens and ANMEs from the Euryarchaeota phylum using a concatenated alignment of a set of 37 conserved marker genes. Lineage Methanomicrobiales* contains four Methanoflorens genomes, five Methanocella genomes, seven Halobacteria genomes, and 29 Methanomicrobiales genomes. (B) The McrABG protein sequence phylogenetic tree (ACR refers to alkyl‐CoM reductase while ECR refers to ethyl‐CoM reductase). Bootstrap values higher than 0.8 are shown with gray squares on tree branches. ANME‐1 and ANME‐2d have been renamed as *Ca*. Methanophagales and *Ca*. Methanoperedenaceae, respectively. Scale bars in gray show the number of substitutions per site. Pink colored branches: *Ca*. Methanophagales (ANME‐1); blue‐colored branches: ANME‐2c; red‐colored branches: ANME‐2a/b; green‐colored branches: *Ca*. Methanoperedenaceae (ANME‐2d); purple‐colored branches: ANME‐2 UWMA‐0185. (C–G) The methane energy metabolisms of ANME‐1, ANME‐2a/b, ANME‐2c, and ANME‐2d from their predicted ancestral genomes as well as ANME‐3 genome, respectively. The methane metabolism gain and loss of the ANMEs' groups can be found in Table S5, whereas some bias may occur because ANMEs' genomes used here are not complete genome from the pure strain. (H) Time tree of the major ANME lineages. The phylogenetic tree was constructed by the sequences of SMC plus 37 conserved protein sequences^8^ using model R10 + C60 + F + G in IQ‐Tree. Molecular dating was calculated by MCMCtree with three different age constraints; that is, the potential fossil evidences of the cell with similar morphology to the Nostocales and Stigonematales (>1.2, >1.7, >2.0 Ga), the potential crown group of oxygenic Cyanobacteria (2.42–2.97 Ga), the predicted origin of the class I methanogen (3.51–4.29 Ga). The tree displayed here is based on the calibration priors >1.7 Ga for the stem lineage of Nostocales, 2.42–2.97 Ga for the crown group of the oxygenic Cyanobacteria (green arrows), and the potential age of class I methanogen 3.51–4.29 Ga.

Phylogenetic positions of ANME‐2 show that they all belong to the order Methanosarcinales and that their evolutionary history is more complicated than that of ANME‐1^4^, ^9^. To date, ANME‐2 has three major clades; that is, ANME‐2a/b, ANME‐2c, and ANME‐2d (Figure 1A). ANME‐2a/b, ANME‐2c, and ANME‐2d are family‐level lineages and cluster relatively close to each other between the methanogen family Methanotrichaceae and Methanosarcinaceae (Figure 1A). Nevertheless, the exact phylogenetic positions of ANME‐2a/b, ANME‐2c, and ANME‐2d are slightly different among the genome and McrABG, as well as 16S rRNA gene trees (Figures 1A,B, S1, and S2). On the genome tree (Figure 1A), ANME‐2a/b clusters with ANME‐2d and a recently discovered anaerobic ethane‐oxidizing archaeal family *Ca*. Argoarchaeaceae (reviewed in Wang et al.^4^). ANME‐2c clusters closely with ANME‐2a/b and ANME‐2d but displays as a deeper branched group with other four families: ANME‐2a/b, ANME‐2d, *Ca*. Argoarchaeaceae, and Methanosarcinaceae. Interestingly, there is one species (ANME‐2 UWMA‐0185, assembled from mid‐Cayman rise vent fluids sample) that clusters next to ANME‐2c in the genome tree, but in the McrABG phylogenetic tree, it places as the deepest branch of the ANME‐2a/b and ANME‐2d clades (Figure 1A,B). Taxonomic classification via GTDB‐tk indicates that ANME‐2 UWMA‐0185 may represent a new family (Table S4). In the genome tree (Figure 1A), ANME‐2 UWMA‐0185 clusters closely with the family ANME‐2c, ANME‐2a/b, and ANME‐2d, suggesting a close evolutionary relationship. As might be expected, the total ANME‐2 families, as well as *Ca*. Argoarchaeaceae and Methanosarcinaceae may have shared one last common ancestor, possibly an early methanogen lineage from the order Methanosarcinales, as indicated in the phylogenetic trees (Figures 1, S1, and S2).

By comparing the reference McrA database and prokaryotic protein sequences from the NCBI genome database, one McrA sequence is found to closely cluster with a previously proposed ANME‐3 McrA sequence^17^. Here, we consider that the genome containing this ANME‐3 McrA‐encoding gene belongs to the ANME‐3 clade (Figure 1A,B). The potential ANME‐3 genome (GLR1503) branches within the family Methanosarcinaceae, and is close to the methanogen genera *Methanosalsum*, *Methanolobus*, *Methanococcoides*, and *Methanohalophilus*, but displays a single branch rather than clusters with other methanogens (Figure 1A). No 16S rRNA gene is obtained from this genome, but the McrABG phylogenetic position displays a topology similar to that of the genome tree, suggesting that McrABG sequences from genome GLR1503 were vertically inherited. Our results indicate that ANME‐3 is a genus‐level lineage that has independently evolved from one methanogen ancestor within the family Methanosarcinaceae (Table S4).

In general, the AOM process is catalyzed by enzymes from the methanogenesis pathway, which typically includes the activation of methane or multicarbon alkanes to methyl‐CoM or alkyl‐CoM via MCR or ACR enzymes, respectively, and then the complete oxidation of methyl‐CoM or alkyl‐CoM to carbon dioxide^2^, ^3^, ^4^, ^9^. The electrons released from methane or alkane oxidation are then transferred through a variety of electron carriers to sulfate‐reducing bacteria (SRB) or directly to other terminal electron acceptors, such as nitrate, nitrite, or metal oxides^9^. ANME‐1 belongs to the short‐chain alkane‐oxidizing archaeal class *Ca*. Syntrophoarchaeia and clusters next to the order *Ca*. Alkanophagales (Figure 1A). The deep branching lineages of the class *Ca*. Syntrophoarchaeia contain genes that code for ACR, a cytoplasmic heterodisulfide reductase (HdrABC), Wood‐Ljungdahl, and beta‐oxidation pathways, F_420_H_2_:quinone oxidoreductase (FQO), and a NADH‐dependent electron transfer system^4^, ^8^. These enzymes enable them to completely oxidize multicarbon alkanes to carbon dioxide. However, the ancestor of ANME‐1 (Figure 1C) gained MCR‐encoding genes, possibly from *Ca*. Methanofastidiosales or *Ca*. Nuwarchaeales, and enabled its methane metabolism. ANME‐1 might have subsequently lost genes coding for the ACR enzyme, acyl‐coenzyme A (CoA)‐related enzymes, and key enzymes for NADH‐dependent electron transfer, such as electron transfer flavoprotein (ETF). These enzymes are critical for the oxidation of multicarbon alkanes, suggesting that the ANME‐1 ancestor has lost the ability of multicarbon alkane oxidation and transformed into a methane oxidizer. Like ANME‐1, all ANME‐2 oxidize methane through methane activation to methyl‐CoM by MCR, complete oxidation of methyl‐CoM to carbon dioxide via the Wood‐Ljungdahl pathway, and most of them perform coenzyme cycling by both cytoplasmic (HdrABC) and membrane‐bound (HdrDE) heterodisulfide reductases along with F_420_H_2_ dehydrogenase (FPO) and other membrane‐bound enzymes^19^. However, most methanogens within the order Methanosarcinales also contain similar enzymes described above, raising the long‐standing question of how ANMEs reverse the methanogenesis pathway or vice versa^9^. Based on our comparative genomic analyses, a common feature of the ANME‐2 clade is that the last common ancestor of ANME‐2a/b, ANME‐2c, and ANME‐2d gained several cytochrome‐encoding genes, which were derived from bacterial or archaeal donors (Figures 1D–F and S3, Table S5). For example, ANME‐2a/b and ANME‐2d gained cytochrome *b*/*b*6 domain‐containing protein‐encoding genes near the gene cluster coding for a membrane‐integral ferredoxin:NAD^+^ oxidoreductase complex (RNF), while ANME‐2a/b and ANME‐2c gained a bacterial type cytochrome *c* protein‐encoding gene. The ANME‐2d also gained potential electron transfer genes, such as genes coding for putative cytochrome *bc*, c554, Rieske‐like domain protein, and ETF. ANME‐3 phylogenetically clusters within the family Methanosarcinaceae and should have been the most recent methanogenesis reversal evolutionary event among all the known ANMEs (Figure 1A). It gained genes coding for the bacterial type cytochrome *c* as well as cytochrome *b*/*b*6 near the gene cluster RNF complex (Figures 1G and S3). ANME‐3 also lost genes that code for the F_420_H_2_ dehydrogenase subunit O (FpoO) and some uncharacterized methanogenesis marker proteins. Based on the above results, we hypothesize that the “methanogenesis‐AOM” transition barriers within the order Methanosarcinales are not high, and the acquisition of genes coding for proteins such as a certain type of cytochrome might enhance the driving force for electron transfer away from the ANME membrane, leading to metabolic reactions toward methane oxidation. Therefore, it can be predicted that novel ANMEs other than ANME‐2 and ‐3 are highly likely to be discovered within the order Methanosarcinales in the future by extensive environmental investigation or laboratory enrichment.

By taking advantage of the HGT event involving the transfer of the chromosome segregation protein (SMC)‐encoding genes from the class Methanomicrobia to the cyanobacterial ancestor^7^, we were able to estimate the origin date of archaeal lineages because an absolute time constraint from Cyanobacteria fossil records can be used to date events within Archaea. In line with the previous reports^7^, ^8^, Cyanobacteria clusters with Methanomicrobia in Euryarchaeota and the high congruence between the SMC phylogenetic tree and the phylogenomic tree of nonphotosynthetic and photosynthetic Cyanobacteria indicates that SMC‐encoding genes were vertically inherited after the HGT to the last common ancestor of the Cyanobacteria and their non‐Cyanobacteria sister lineages (Figure 1H). Based on the constraints from the predicted crown group date of oxygenic Cyanobacteria, the potential Nostoc‐like fossils and the predicted origin date of the class I methanogen (File S1), we predict that the major ANME lineages may have originated around 2.66 to 1.88 Ga, with the most widely distributed ANME‐1 and ANME‐2a/b/c lineages originating at ~2.66 to 2.18 Ga (Table 1). The origin dates of ANME‐1 and ANME‐2a/b/c are possibly correlated with the Great Oxygenation Event (GOE) and Huronian Glaciation^20^. It was hypothesized that the Huronian Glaciation was triggered by the consumption of carbon dioxide via physical erosion and chemical weathering from tectonic activity or GOE because the oxygen generated by Cyanobacteria oxidized the atmospheric methane that had been produced by methanogen and accumulated on the previously anaerobic Earth^21^, ^22^. Here, by adding new evidence to the origin date of ANMEs, we suggest that the AOM process may also have contributed to the Huronian Glaciation.

**Table 1:** Molecular clock age estimations for the emergence of different ANME groups.

	Stem ANMEs' age estimations (Ga)		
Name	>1.2	>1.7	>2.0
ANME‐1	2.24 (1.87–2.98)	2.35 (1.73–2.91)	2.43 (1.90–3.01)
ANME‐2a/b	2.18 (1.80–2.61)	2.20 (1.76–2.60)	2.19 (1.75–2.68)
ANME‐2c	2.65 (2.24–3.04)	2.66 (2.25–3.04)	2.66 (2.23–3.08)
ANME‐2d	1.89 (1.50–2.35)	1.88 (1.45–2.32)	1.88 (1.41–2.41)

Three calibration time points were used: the predicted emergence time of class I methanogen (3.51–4.29 Ga), the predicted crown group of oxygenic Cyanobacteria (2.42–2.97 Ga), as well as three independent predicted ages (>1.2, >1.7, and >2.0 Ga) of the pontential fossil evidences of akinete‐forming cells for the Nostocales and Stigonematales groups. The stem ANMEs' age estimations are indicated as median ages and 95% confidence interval in Ga based on the three calibration age sets described above, as displayed in three separate columns in the table. ANME, anaerobic methane‐oxidizing archaea.

In summary, the first methanogen may have originated in the late Hadean or early Archaean eon and could have used hydrogen and methylated compounds to generate methane^8^. After the introduction of tetrahydromethanopterin *S*‐methyltransferase (MTR) in the early‐mid Archaean, methanogens were able to carry out methanogenesis through hydrogen oxidation and carbon dioxide reduction^23^. The ability to produce methane via carbon dioxide reduction should have significantly increased the amount of methane generated and caused an increase in the Earth's surface temperature. After the origin of oxygenic Cyanobacteria as well as the GOE, the oxidation rates of sulfur compounds to sulfate might have increased partially by the rising oxygen concentration and stimulated the expansion of sulfate‐reducing bacteria, which might have triggered the origin of ANMEs in the late Archaean to early Proterozoic eon. The GOE, along with the AOM process conducted by ANMEs, potentially caused Huronian Glaciation on Earth and the huge geological and ecological influences hereafter.

## AUTHOR CONTRIBUTIONS

Yinzhao Wang designed the research, performed the analyses, and wrote the paper. Fengping Wang provided guidance and wrote the paper. Jialin Hou and Ruize Xie performed the evolutionary analyses. Liuyang Li, Yaoxun Hu, Zhenbo Lv, and Hungchia Huang performed the genomic analyses.

## ETHICS STATEMENT

Not applicable.

## CONFLICT OF INTERESTS

The authors declare that they have no competing interests.

## Supporting information

Supporting Information.

Supporting Information.

Supporting Information.

## Data Availability

All genomic data can be downloaded from the NCBI database (www.ncbi.nlm.nih.gov/assembly/).
