# Supplementary material for: The late Archaean to early Proterozoic origin and evolution of anaerobic methane‐oxidizing archaea
Source: mLife. 2022 Mar 30;1(1):96–100. doi: 10.1002/mlf2.12013 (PMC10989977; doi:10.1002/mlf2.12013)
Supplement: Supplementary file 2 — Supporting Information. [file MLF2-1-96-s003.docx]

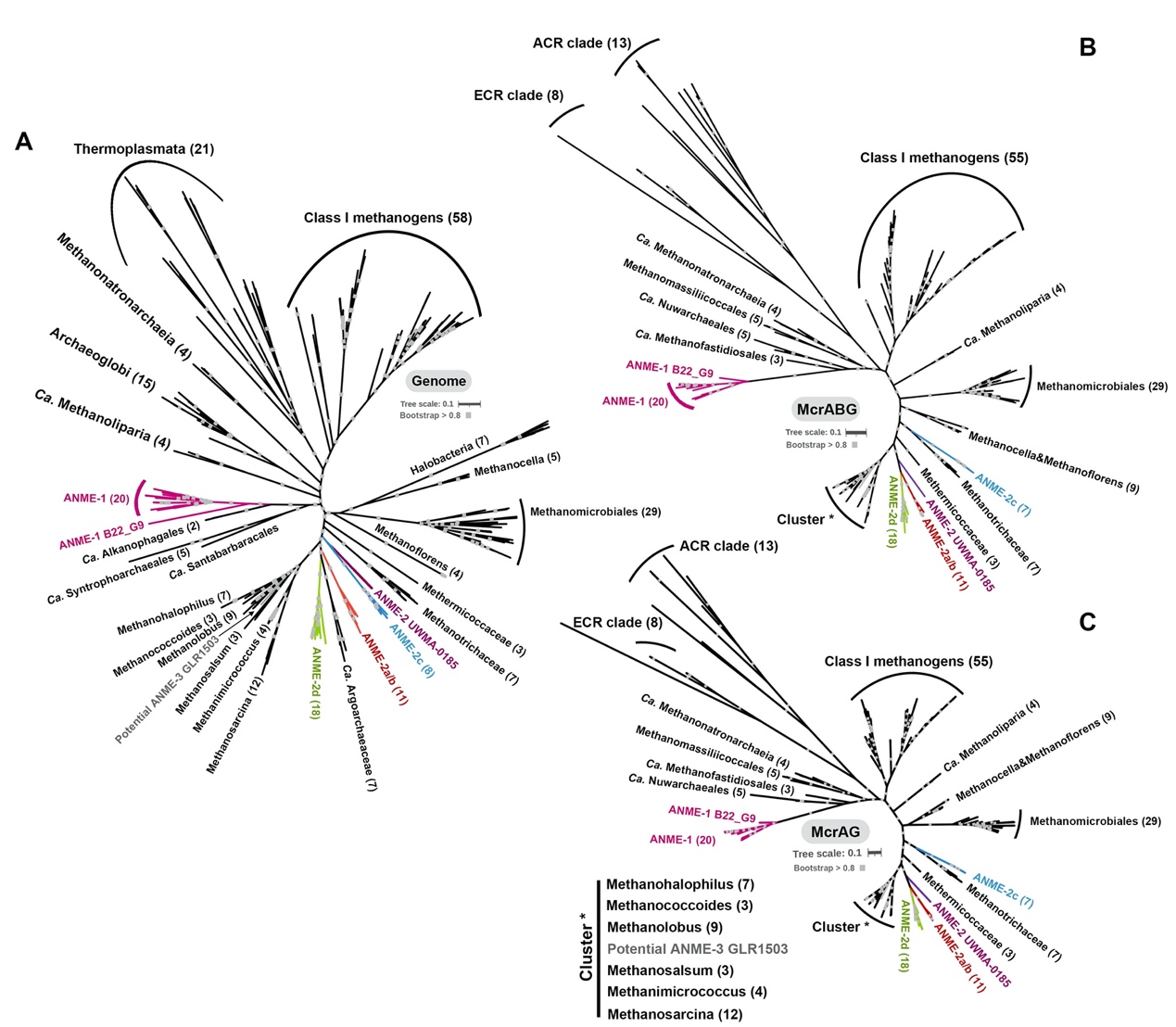


Supplementary figure 1. The lineage-expanded phylogenetic trees of methanogens and ANMEs from the Euryarchaeota superphylum. (A) Phylogenomic tree using a set of 37 conserved marker genes and (B) phylogenetic tree with the McrABG protein sequences, and (C) phylogenetic tree with the more conserved McrAG protein sequences. The bootstrap values higher than 0.8 are shown with gray square on tree branches.


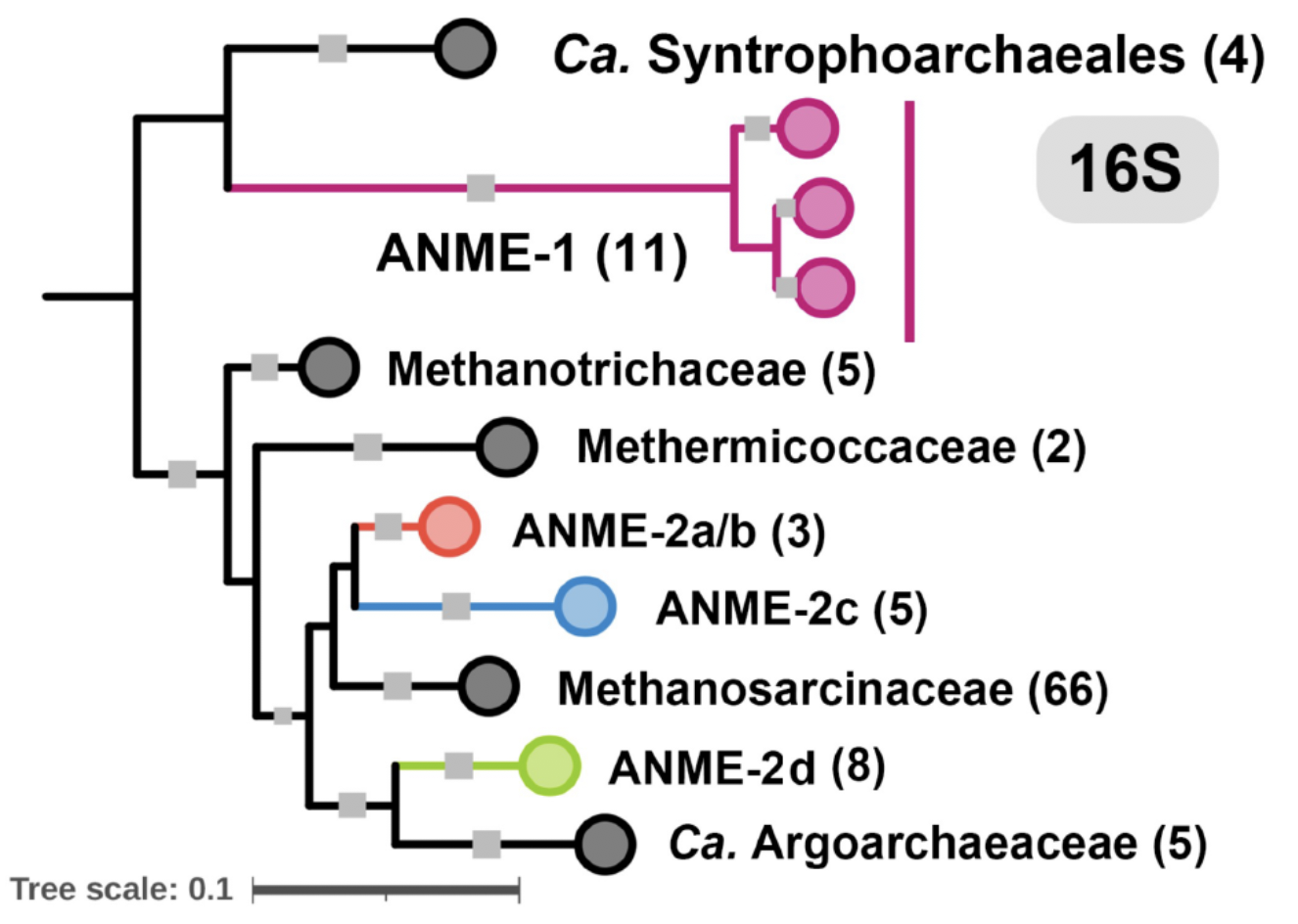


Supplementary figure 2. The 16S rRNA gene phylogenetic tree of the class *Ca.* Syntrophoarchaeia and order Methanosarcinales. Phylogenetic alignment was based on MAFFT and then filtered with trimAl, and the trees were built by the IQ-Tree using SH-approximate-likelihood ratio test implemented with 1000 bootstrap replicates with bootstraps higher than 0.8 shown with gray square on tree branches.


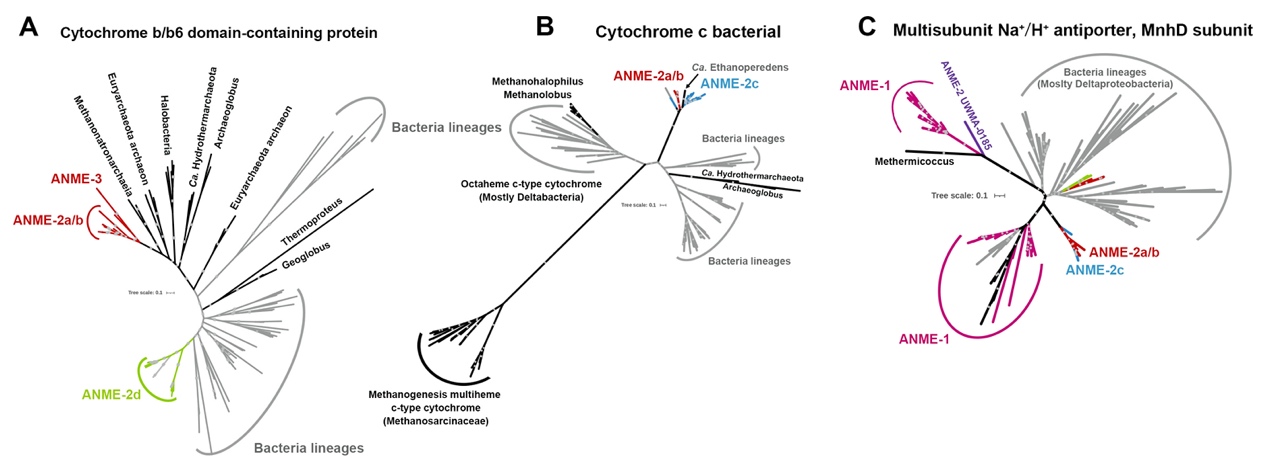


Supplementary figure 3. Phylogenetic trees of potential horizontally transferred cytochrome-encoding genes from ANMEs. (A) Phylogenetic trees of cytochrome b/b6 domain-containing protein, (B) Cytochrome c bacterial and (C) one potential multisubunit Na^+^/H^+^ antiporter MnhD subunit sequences. These genes might be processed by some ANME lineages but not conserved in methanogens genomes. Bootstrap values higher than 0.8 are shown with gray square on tree branches. Based on their topologies, it is highly likely that these genes within ANMEs were horizontally transferred from bacterial or archaeal donors.
