# Supplementary material for: The late Archaean to early Proterozoic origin and evolution of anaerobic methane‐oxidizing archaea
Source: mLife. 2022 Mar 30;1(1):96–100. doi: 10.1002/mlf2.12013 (PMC10989977; doi:10.1002/mlf2.12013)
Supplement: Supplementary file 3 — Supporting Information. [file MLF2-1-96-s002.docx]

**Supplementary file 1**

**Materials and methods**

**Prokaryotic genomes from the NCBI database**

All the prokaryotic genomes were downloaded from the NCBI prokaryotic genome database. Then, the McrA/B/G sequences from the NCBI protein nr database were also downloaded, and local McrA/B/G databases by DIAMOND version 0.8.28.90 (1) were constructed. We then searched the potential ANME McrA/B/G sequences from the downloaded prokaryotic genomic datasets with DIAMOND with parameters -e 1e-20 --query-cover 75, and McrA/B/G sequences with the best hits to the known ANME McrA/B/G were selected and low-quality genomes (Completeness < 70%, Redundancy > 7%) were discarded (Supplementary table 1).

**Phylogenetic analyses**

For phylogenomic analysis, representative euryarchaeal reference genomes (Supplementary Table 3, totally 208 genomes from the class I methanogens, Thermoplasmata, Methanonatronarchaeia, Archaeoglobi, *Ca.* Methanoliparia, *Ca.* Syntrophoarchaeia, and the class II methanogens) were also downloaded from the NCBI prokaryotic genome database. These reference genomes and the 60 ANME genomes (21 ANME-1, 38 ANME-2, 1 ANME-3) were used to construct a phylogenomic tree based on a concatenated alignment of a set of 37 marker genes (Supplementary Table 2). The 37 marker protein sequences from the genomes described above were aligned using the MAFFT algorithm version 7.313 (2) with parameters --ep 0 –genafpair --maxiterate 1000 and then filtered with trimAl version 1.4.rev2 (3) with parameter -automated1, and all 37 marker genes were concatenated. The phylogenomic tree was built by IQ-Tree version 1.6.6 (4) with model R8+C40+F+G and an ultrafast bootstrap value of 1000 and was rooted in the class I methanogens. For the phylogenetic analysis of 16S rRNA genes and McrABG, the sequences from the lineages containing ANMEs (*Ca.* Syntrophoarchaeia and the class II methanogens) were retrieved from the genomes in the present study, then aligned by MAFFT algorithm version 7.313 (2), filtered by trimAl version 1.4.rev2 (3) and phylogenetic trees were constructed by IQ-tree version 1.6.6 (4).

**Evolutionary analysis and divergence time estimation**

Genomes were predicted with Prodigal version 2.6.3,(5) and the predicted ORFs were annotated in the eggNOG database.(6) The comparative genomic analysis was conducted by OrthoFinder (7) with default parameters. A total of 12,045 orthogroups from 123 representative genomes were obtained from OrthoFinder and transformed to the presence and absence data. The 123 representative genomes contain ANMEs and class II methanogens, with 86 genomes > 90%, 18 genomes within the range 80%~90%, 17 genomes within the range 70%~80%, two genomes within the range 60%~70% but evolutionary important. Ancestral family gene sets were inferred using the program COUNT.(8) We used all three algorithms (Dollo, Wagner and Posteriors) provided by COUNT to calculate the gene sets at each node, and genes predicted by either two algorithms are considered valid, then used for further comparison (Supplementary Table 5). For genes that considered important for methane energy metabolism, a manual check for the presence and absence from ANME genomes were conducted by a blastp search on NCBI website, and three potential cytochrome genes that processed by ANMEs possibly from HGT events were collected and phylogenetic trees were constructed using IQ-Tree version 1.6.6 with best-fitting model with either C40 or C60 model and an ultrafast bootstrap value of 1000. Molecular dating was conducted by Bayesian estimation of species divergence times using soft fossil constraints MCMCTree version v4.9c (9) with WAG model. In total three different age combinations were used for the ANME origin date estimation: the crown group of oxygenic Cyanobacteria (2.42 to 2.97 Ga), the potential fossil evidences of the cell with similar morphology to the Nostocales and Stigonematales (>1.2, >1.7, and >2.0 Ga) as well as the predicted origin of the class I methanogen, i.e., 3.51-4.29 Ga.(10-18) The phylogenetic tree of the SMC plus 37 conserved protein sequences (19) was constructed by model R10+C60+F+G in IQ-Tree version 1.6.6 for MCMCtree configuration.

**References**

1. Buchfink B, Xie C, Huson DH. Fast and sensitive protein alignment using DIAMOND. Nat Methods. 2015;12:59.

2. Katoh K, Standley DM. MAFFT multiple sequence alignment software version 7: improvements in performance and usability. Mol Biol Evol. 2013;30:772-80.

3. Capella-Gutiérrez S, Silla-Martínez JM, Gabaldón T. trimAl: a tool for automated alignment trimming in large-scale phylogenetic analyses. Bioinformatics. 2009;25:1972-3.

4. Nguyen LT, Schmidt HA, von Haeseler A, Minh BQ. IQ-TREE: a fast and effective stochastic algorithm for estimating maximum-likelihood phylogenies. Mol Biol Evol. 2015;32:268-74.

5. Hyatt D, Chen GL, LoCascio PF, Land ML, Larimer FW, Hauser LJ. Prodigal: prokaryotic gene recognition and translation initiation site identification. BMC Bioinformatics. 2010;11:119.

6. Huerta-Cepas J, Forslund K, Coelho L P, Szklarczyk D, Jensen LJ, Von Mering C. Fast genome-wide functional annotation through orthology assignment by eggNOG-mapper. Mol Biol Evol. 2017;34:2115-22.

7. Emms DM, Kelly S. OrthoFinder: phylogenetic orthology inference for comparative genomics. Genome Biol. 2019;20:1-14.

8. Csűös M. Count: evolutionary analysis of phylogenetic profiles with parsimony and likelihood. Bioinformatics. 2010;26:1910-2.

9. Dos Reis M. Notes on the birth–death prior with fossil calibrations for Bayesian estimation of species divergence times. Proc Royal Soc B. 2016;371:20150128.

10. Horodyski RJ, Donaldson JA. Microfossils from the middle Proterozoic Dismal Lakes groups, arctic Canada. Precambrian Res. 1980;11:125-59.

11. Amard B, Bertrand-Sarfati J. Microfossils in 2000 Ma old cherty stromatolites of the Franceville Group, Gabon. Precambrian Res. 1997;81:197-221.

12. Sheridan PP, Freeman KH, Brenchley JE. Estimated minimal divergence times of the major bacterial and archaeal phyla. Geomicrobiol J. 2003;20:1-14.

13. Sánchez-Baracaldo P. Origin of marine planktonic cyanobacteria. Sci Rep. 2015;5:1-10.

14. Sánchez-Baracaldo P, Bianchini G, Wilson JD, Knoll AH. Cyanobacteria and biogeochemical cycles through Earth history. Trends Microbiol. 2021;doi.org/10.1016/j.tim.2021.05.008.

15. Shih PM, Hemp J, Ward LM, Matzke NJ, Fischer WW. Crown group Oxyphotobacteria postdate the rise of oxygen. Geobiology. 2017;15:19-29.

16. Wolfe JM, Fournier GP. Horizontal gene transfer constrains the timing of methanogen evolution. Nat Ecol Evol. 2018;2:897.

17. Boden JS, Konhauser KO, Robbins LJ, Sánchez-Baracaldo P. Timing the evolution of antioxidant enzymes in cyanobacteria. Nat Commun. 2021;12:1-12.

18. Fournier GP, Moore KR, Rangel LT, Payette JG, Momper L, Bosak T. The Archean origin of oxygenic photosynthesis and extant cyanobacterial lineages. Proc Royal Soc B. 2021;288:20210675.

19. Wang Y, Wegener G, Williams TA, Xie R, Hou J, Tian C, et al. A methylotrophic origin of methanogenesis and early divergence of anaerobic multicarbon alkane metabolism. Sci Adv. 2021;7:eabj1453.
